# A comparison of genotyping-by-sequencing analysis methods on low-coverage crop datasets shows advantages of a new workflow, GB-eaSy

**DOI:** 10.1186/s12859-017-2000-6

**Published:** 2017-12-28

**Authors:** Daniel P. Wickland, Gopal Battu, Karen A. Hudson, Brian W. Diers, Matthew E. Hudson

**Affiliations:** 1 Department of Crop Sciences, University of Illinois at Urbana-Champaign, Urbana, IL 61801 USA; 2 Illinois Informatics Institute, University of Illinois at Urbana-Champaign, Urbana, IL 61801 USA; 3 HudsonAlpha Institute for Biotechnology, 601 Genome Way, NW, Huntsville, AL 35806 USA; 4 USDA-ARS Crop Production and Pest Control Research Unit, 915 West State Street, West Lafayette, IN 47907 USA

**Keywords:** GBS, WGS, Bioinformatics pipelines, Variant calling, Soybean, Crops

## Abstract

**Background:**

Genotyping-by-sequencing (GBS), a method to identify genetic variants and quickly genotype samples, reduces genome complexity by using restriction enzymes to divide the genome into fragments whose ends are sequenced on short-read sequencing platforms. While cost-effective, this method produces extensive missing data and requires complex bioinformatics analysis. GBS is most commonly used on crop plant genomes, and because crop plants have highly variable ploidy and repeat content, the performance of GBS analysis software can vary by target organism. Here we focus our analysis on soybean, a polyploid crop with a highly duplicated genome, relatively little public GBS data and few dedicated tools.

**Results:**

We compared the performance of five GBS pipelines using low-coverage Illumina sequence data from three soybean populations. To address issues identified with existing methods, we developed GB-eaSy, a GBS bioinformatics workflow that incorporates widely used genomics tools, parallelization and automation to increase the accuracy and accessibility of GBS data analysis. Compared to other GBS pipelines, GB-eaSy rapidly and accurately identified the greatest number of SNPs, with SNP calls closely concordant with whole-genome sequencing of selected lines. Across all five GBS analysis platforms, SNP calls showed unexpectedly low convergence but generally high accuracy, indicating that the workflows arrived at largely complementary sets of valid SNP calls on the low-coverage data analyzed.

**Conclusions:**

We show that GB-eaSy is approximately as good as, or better than, other leading software solutions in the accuracy, yield and missing data fraction of variant calling, as tested on low-coverage genomic data from soybean. It also performs well relative to other solutions in terms of the run time and disk space required. In addition, GB-eaSy is built from existing open-source, modular software packages that are regularly updated and commonly used, making it straightforward to install and maintain. While GB-eaSy outperformed other individual methods on the datasets analyzed, our findings suggest that a comprehensive approach integrating the results from multiple GBS bioinformatics pipelines may be the optimal strategy to obtain the largest, most highly accurate SNP yield possible from low-coverage polyploid sequence data.

## Background

The development of second-generation, short-read sequencing has revolutionized biological research, agriculture and medicine, enabling innovations such as genomic selection to raise crop yields and precision medicine to diagnose and treat disease. The single-nucleotide polymorphisms (SNPs) identified by high-throughput sequencing serve as markers for association between genotypes and phenotypes. Whole-genome sequencing can identify millions of SNPs, but for many applications involving genetic linkage, such high densities of markers are unnecessary. Reduced-representation approaches involve sequencing a subset of locations spread throughout the genome to reduce genome complexity and rapidly genotype samples using SNP markers. The earliest reduced-representation sequencing method, restriction site associated DNA (RAD) sequencing, used restriction enzymes to divide the genome into sheared DNA fragments, which were size fractionated and then sequenced on next-generation sequencing platforms [1–3]. RAD sequencing remains the method of choice for biological diversity applications in which reference genomes are not available. In this and similar methods, each sample is assigned a unique barcoded adapter for multiplexed sequencing in a single Illumina flow-cell lane, thereby increasing the number of samples under investigation and reducing financial costs. Although this method works well on crops such as soybean [4], the large amount of high-quality DNA required for the size selection step, and consequent higher DNA preparation costs, makes RAD sequencing unsuitable for routine use in plant breeding.

Genotyping-by-sequencing (GBS), a simplified reduced-representation sequencing approach [5], has gained popularity in crop research and plant breeding for high throughput, low-cost genotyping. It has been applied to projects ranging from genomic selection to gene mapping to genome-wide association studies in numerous crop species [6–10]. Like RAD sequencing, GBS relies on restriction enzymes to generate a reduced representation of the genome for sequencing. However, the GBS library preparation protocol involves fewer steps than RAD sequencing, requires less DNA, and lacks a size selection step [5]. In GBS, DNA samples are digested and ligated to barcoded adapters in single wells, pooled, and then enriched by PCR. An important development in GBS was the incorporation of a two-enzyme digestion into the protocol [11].

In contrast to the relatively simple and straightforward library preparation, GBS and RAD sequencing data analysis is complicated by the nature of the random location, reduced-representation approach. The data analysis requires individual alignment of the reads, generates a large proportion of missing data, and requires several statistical assumptions to be made in order to call variants. Bioinformatics software packages and workflows have been developed to provide the architecture for analysis of reduced-representation sequencing data [12–14]. Several of these platforms utilize the same tools and algorithms commonly applied to whole-genome sequence data, while others utilize algorithms developed specifically for GBS and RAD sequencing. Although designed to facilitate and simplify data processing, these GBS pipelines nevertheless can be difficult for non-specialist researchers such as plant breeders to install or implement. Issues include high levels of complexity, requirements for additional libraries or uncommon packages, or additional processing steps outside of the pipelines. A different approach, TASSEL / TASSEL-GBS [15, 16], provides an all-in-one desktop software package that is easy to install and use, and performs both GBS data processing and genetic analysis using the resources of a stand-alone PC. However, while this software is widely adopted in cereal genetics, it was optimized for use in maize, and uses heuristics such as the reduction of reads to tags before alignment to enable reasonable run times on PC hardware. These heuristics are less clearly advantageous in recently polyploid species; for this reason, others (e.g. [14]) have developed different approaches for crops such as soybean. Finally, the all-in-one software package approach means that users cannot themselves modify TASSEL-GBS to accommodate new sequencing technology or other software packages.

More recently, known segregating sites from pan-genome data have been shown to substantially improve accuracy and yield from reduced-representation sequencing [17]; however, for other crops such as soybean and many others important for food production, population-level diversity is not yet sufficiently well characterized at the whole-genome level, and better tools to identify SNPs ab initio are still needed. In addition, recently polyploid genomes such as soybean [18] present a complication to the performance of alignment and variant calling for all forms of reduced-representation sequencing. This may influence the performance of different approaches relative to more straightforward diploid genomes.

Here we present GB-eaSy, a GBS bioinformatics pipeline that efficiently incorporates widely used genomics tools, parallelization and automation to increase the accuracy, efficiency and accessibility of GBS analysis. GB-eaSy has been specifically developed to be straightforward to install and use on typical UNIX / HPC hardware, to contain readily updateable public software where possible, and to match or exceed the performance of current GBS SNP-calling methods used on soybean or other complex, repetitive and recently polyploid genomes. It can process reduced-representation data from any organism with a reference genome. We compared the performance of GB-eaSy to four other GBS bioinformatics data analysis platforms using low-coverage Illumina sequence data from three soybean populations. GB-eaSy rapidly and accurately identified the greatest number of SNPs across all three populations, with SNP calls in close agreement with whole-genome sequencing of selected lines.

## Methods

### Samples

GBS libraries were constructed from three soybean populations (Table 1). Population 1 consisted of 378 F2 lines resulting from a cross between the accession Prize and an NMU-mutagenized individual from the reference genotype Williams 82. Population 2 contained 391 F2 individuals from a cross between two breeding lines. Finally, Population 3 consisted of 81 unrelated accessions (with 2–4 replications) that form an association panel.

**Table 1:** GBS library data for the three populations analyzed in this study

	Population 1	Population 2	Population 3
Description	F2 from cross between Prize and mutagenized Williams 82	F2 from cross between two breeding lines	81 unrelated lines
Number of samples	378	391	200
Sequencer	Illumina HiSeq2500	Illumina HiSeq4000	Illumina HiSeq2500
Read length	100 bp	100 bp	100 bp
Number of reads	234,574,472 (single-end)	392,001,642 (single-end)	247,063,538 (single-end)
Average depth per sequenced base	1.87 reads	3.63 reads	4.47 reads
Average percent of genome covered by at least 1 read	2.29	2.02	2.35
Average percent of genome covered by at least 2 reads	1.08	1.42	1.71

DNA was extracted using the CTAB method [19] except for the Prize x NMU-mutagenized Williams 82 population (Population 1), which used the E-Z 96 Plant DNA kit (Omega Bio-Tek, Norcross, GA). All libraries were sequenced at low coverage typical of plant breeding experiments, with coverage varying from 1.87× to 4.47×

### GBS library preparation

GBS libraries were prepared according to the two-enzyme protocol described in [6] with minor modifications (kindly provided by Dr. P. Brown, UC Davis). Two-enzyme pairs (HindIII-MseI and HindIII-BfaI) were used to achieve a balanced representation of HindIII cut sites. In brief, restriction and ligation were carried out simultaneously, followed by PCR amplification. First, 5 μl of DNA (25–50 ng/μl, 125-250 ng total) from each sample was pipetted into its own well on a 384-well plate that contained restriction-ligation master mix. The master mix in each well consisted of 2.5 μl 10× NEB CutSmart buffer (final concentration 1×), 2.5 μl 10 mM dATP (final concentration 1 mM), 0.1 μl (2 U) HindIII, 0.2 μl MseI or BfaI, 0.1 μl concentrated T4 DNA ligase (40 U), 0.5 μl each of 10uM adapters, and 14.1 μl molecular biology-grade water. The barcoded “rare adapters” were designed to anneal to the cut HindIII site, while the non-barcoded “common adapters” annealed to the cut MseI or BfaI site.

Covered with foil, the 384-well plates underwent digestion and ligation in the thermocycler at 37 °C for 1 min, 25 °C for 1 min, repeated 100 times. Next, 8 μl from each well was pooled into a 1.5 mL microfuge tube, cleaned using Agencourt AMPure XP beads (Beckman Coulter Life Sciences, Indianapolis, Indiana, USA), dried, and suspended for PCR amplification in a solution of Phusion Master Mix (NEB, Ipswich, MA). PCR settings for amplification were 98 °C for 30s, 15 cycles (98 °C for 10s, 68 °C for 30s, 72 °C for 30s), 72 °C for 5 m, followed by 4 °C until sample recovery. Next, AMPure cleanup was repeated, and the resulting library was evaluated on a Bioanalyzer 2100 (Agilent, Santa Clara, CA) using a DNA7500 chip to assess amplification success, fragment size, and DNA concentration. Finally, each library was diluted to 10 nM DNA in LIB buffer (10 mM Tris-HCL (EB) w/ 0.05% Tween-20) and run on either an Illumina HiSeq2500 or HiSeq4000 using the HiSeq SBS sequencing kit version 4 at the Roy J. Carver Biotechnology Center at the University of Illinois at Urbana-Champaign.

### GBS data analysis platforms

#### Tassel-GBS

TASSEL-GBS was developed to assign SNP genotypes from GBS data in a time- and storage-efficient manner [16] (Table 2). Unlike SNP calling for whole-genome data, which involves first aligning all reads to the reference genome and then calling SNPs, TASSEL-GBS dramatically reduces computational demands by consolidating reads into a master “tag list” containing the unique sequences. This tag list is then aligned to a reference genome. For species lacking a reference genome, the consensus allele at each position is considered the reference allele. Variant identification in the TASSEL5GBSv2 pipeline (https://bitbucket.org/tasseladmin/tassel-5-source/wiki/Tassel5GBSv2Pipeline) consists of two main steps: SNP discovery and production SNP calling. In SNP discovery, TASSEL-GBS determines SNPs and SNP coverage within each tag for each sample and outputs the results to a database. In production SNP calling, SNP genotypes in each sample are output. Each step is performed internally with TASSEL-GBS plugins, except alignment, which is carried out externally using software such as BWA-MEM [20]. Prior to running TASSEL, we removed adapter sequence from the reads using cutadapt [21] after finding that adapter contamination severely impaired the accuracy of TASSEL-GBS SNP calls relative to the other methods.

**Table 2:** Major steps of the 5 GBS workflows analyzed

	TASSEL-GBS	IGST	Fast-GBS	Stacks	GB-eaSy
Demultiplex reads	GBSSeqToTagDBPlugin, TagExportToTagDBPlugin	Sabre	Sabre	process_radtags	GBSX
Trim adapters	cutadapt*	trimAdaptor3.py	cutadapt	process_radtags	GBSX
Align to reference	bwa-mem*	bwa-aln	bwa-mem	bwa-mem*	bwa-mem
Call SNPs	DiscoverySNPCallerPluginV2, ProductionSNPCallerPluginV2	SAMtools/BCFtools	Platypus	pstacks, cstacks, stacks, populations	BCFtools

Each workflow uses a different series of tools to carry out read demultiplexing, adapter trimming, alignment to the reference genome, and SNP calling

*step performed manually outside the workflow

### Stacks

Stacks is a software package developed for RAD sequencing that identifies SNPs and calculates population statistics from any restriction enzyme-based, reduced-representation sequence data [12] (Table 2). After demultiplexing and cleaning the sequenced reads, Stacks assembles loci from each sample (with or without a reference genome) and groups together loci across samples to construct a catalog. Comparison between the catalog and loci from each sample allows inference of SNPs and genotypes. Optional additional steps include creation of genetic maps and calculation of population statistics. Like TASSEL-GBS, each step except alignment (here performed by BWA-MEM) uses the software’s internal algorithms.

### IGST

IGST (IBIS Genotyping by Sequencing Tools) processes GBS data by implementing several popular genomic software tools connected by Perl and Python scripts [13] (Table 2). After setting up a predefined directory structure and naming input files according to a specific convention, the user issues a single command that runs the entire pipeline. IGST demultiplexes and cleans barcoded reads using Sabre (https://github.com/najoshi/sabre), aligns demultiplexed reads to the reference genome using BWA-ALN [22], converts the aligned sequences to BAM format using SAMtools [23], and identifies SNPs using SAMtools and BCFtools [23]. The resulting SNP calls are filtered by VCFtools [24].

### Fast-GBS

Fast-GBS follows a strategy similar to IGST but employs a different alignment algorithm, a different variant caller, and a bash script that runs each software program [14] (Table 2). As with IGST, the user must set up a predefined directory structure and name files according to a specific convention before inputting a single command to run the workflow. This pipeline demultiplexes reads using Sabre, trims and cleans reads using Cutadapt, aligns reads to the reference genome using BWA-MEM, and calls variants using Platypus [25]. As a haplotype-based variant caller, Platypus identifies single-allele SNPs as well as compound SNPs consisting of short strings of adjacent alleles. To facilitate comparisons with the other pipelines, we used the VariantsToAllelicPrimitives script within the Genome Analysis Toolkit [26] to deconvolute the multi-allelic SNPs into individual allelic primitives, as recommended by [27].

### GB-eaSy

The GB-eaSy pipeline developed for this project consists of a Bash shell script that executes several bioinformatics software programs in a parallel UNIX / Linux environment. This workflow requires a reference genome and is compatible with both single- and paired-end Illumina reads. Its name derives from its straightforward, transparent implementation of GBS variant calling; GB-eaSy is appropriate for users without extensive command-line expertise as well as for experienced bioinformaticians who may choose to modify any step of the script. GB-eaSy implements the same well-tested and regularly updated tools commonly adopted in whole-genome sequencing. In contrast to some GBS pipelines, GB-eaSy does not require the user to follow strict instructions regarding directory structure or file names; instead, the Bash script performs these steps automatically. The GB-eaSy shell script, a walkthrough of each command, and a tutorial using sample data are hosted at https://github.com/dpwickland/GB-eaSy.

Before starting the pipeline, the user modifies a parameters file with settings customized for their GBS project (e.g. path to raw sequencer output file, path to barcodes file, number of CPU cores to use). The user then issues a single command to execute the pipeline. The first step of GB-eaSy uses the software GBSX [28] to demultiplex reads and trim adapter sequences based on a user-created barcodes file containing the short barcode sequences that uniquely identify each sample; for our study, we modified the GBSX script (GBSX.jar) to include the HindIII cut site, which was not supported initially. Next, demultiplexed reads are aligned to the reference genome using BWA-MEM; GB-eaSy hastens this alignment step by processing read files in parallel using GNU Parallel [29]. After alignment, BCFtools is used to create a pileup of read bases from which it calls SNPs. This SNP-calling step uses GNU Parallel to process each entry in the reference genome file (e.g. each chromosome, each scaffold) on its own CPU core, greatly increasing the efficiency of SNP identification. Finally, the output VCF file is filtered by VCFtools according to a user-specified minimum read depth (Table 2).

### Whole-genome sequencing

To validate the output from the GBS pipelines, Illumina whole-genome sequence (WGS) data was obtained (experimentally in the case of Prize for Population 1 and the case of LG12 for Population 2, or from the data obtained by [30] for four lines of the soybean NAM association panel for Population 3) for comparison of GBS and WGS SNP calls (Table 3). As with the GBS pipelines, WGS reads were aligned to the reference genome using the software BWA-MEM. However, variant calling on the WGS datasets was carried out with GATK HaplotypeCaller, a software not used by any of the GBS pipelines, to provide independent assessment of GBS SNP call accuracy.

**Table 3:** WGS library data for six lines

	Prize	LG12	Magellan	Maverick	Prohio	Skylla
Population of origin	Population 1	Population 2	Population 3	Population 3	Population 3	Population 3
Read length	100 bp	150 bp	150 bp	150 bp	150 bp	150 bp
Number of reads	130,404,160 (paired-end)	43,756,742 (paired-end)	12,880,066 (paired-end)	19,038,600 (paired-end)	34,177,159 (paired-end)	23,190,927 (paired-end)
Coverage (LN / G)	13.65	6.87	2.02	2.99	5.37	3.64
Percent of genome covered by at least 1 read	98.67	97.76	74.38	94.06	98.36	96.16
Percent of genome covered by at least 2 reads	98.31	97.04	73.03	85.18	97.27	90.36

*Prize and LG12 were also included in GBS Populations 1 and 2, respectively. Magellan, Maverick, Prohio and Skylla were included in GBS Population 3. Coverage was computed as the product of read length and number of reads, divided by genome size*

### Pipeline comparisons

The five GBS pipelines and the WGS pipeline described above were run with the following parameters to make the analysis as equivalent as possible between workflows: minimum read length of 80 bases after adapter and barcode trimming, minimum base quality of 20 and minimum mapping quality of 20 for variant calling (corresponding to a 1 in 100 chance of an incorrect base call or mapping call, respectively), and identification of SNPs only (no indels). Other parameters were set at default values. The software package VCFtools was then used to remove SNP calls supported by less than 2 reads (i.e. minimum depth of 2 reads) to increase the reliability of distinguishing homozygous from heterozygous genotypes (note that our lowest coverage dataset has an average depth per sequenced base of 1.87×). Recent versions* of component software packages and commands were used for each pipeline, with the following exceptions: for IGST, commands were drawn from SAMtools version 0.1.18 and Picard version 1.119 because the IGST workflow was incompatible with later versions. Finally, 11 CPU cores were used at any steps that carried an option for parallelization. In-house scripts, BCFtools and VCFtools were used to compute and compare the number of chromosomal SNPs identified by the pipelines and to calculate missing data values. All programs were run on a Linux server with two Intel® Xeon® X5650 processor chips, each with six CPU cores, and 48 GB RAM.

GNU parallel 20,170,122.

JAVA 1.8.0_121.

Picard 2.10.0.

BWA 0.7.15-r1140.

Platypus 0.8.1.

TASSEL 5.0, build April 6, 2017.

VCFtools 0.13.

GBSX_v1.3.

SAMtools/BCFtools 1.5.

Cutadapt 1.12.

Stacks 1.46.

## Results

### GBS SNP calls and their agreement with WGS SNP calls

We compared the SNP calls within and between pipelines on three different populations. Populations 1 and 2 were each 384-well plates used to sequence populations of F2 individuals chosen to mimic mapping populations or breeding studies, while Population 3 was a set of 81 diverse lines, again replicated across a 384 well plate, that can be used as a GWAS diversity panel [30]. Population 1 was derived from a cross between Prize (a US-adapted cultivar) and Williams 82 (the target of the reference genome project [18]), while Population 2 was derived from a cross between two breeding lines that should be equally distant from the reference genome. After preparing GBS libraries and obtaining low-coverage Illumina sequence data (ranging from 1.87 to 4.47× depth per sequenced base), we called SNPs using the five pipelines and computed the total number of SNPs identified and the number of SNPs shared between pipelines. In addition, we compared the GBS SNP calls to WGS SNP calls of selected lines to calculate the SNP concordance and allelic concordance between GBS and WGS. The analysis excluded indels to simplify comparisons among the methods (some methods call only SNPs) and to focus on SNPs, which are the markers of choice in most breeding projects. All SNPs were called relative to the Williams 82 soybean reference genome.

**Fig. 1:**
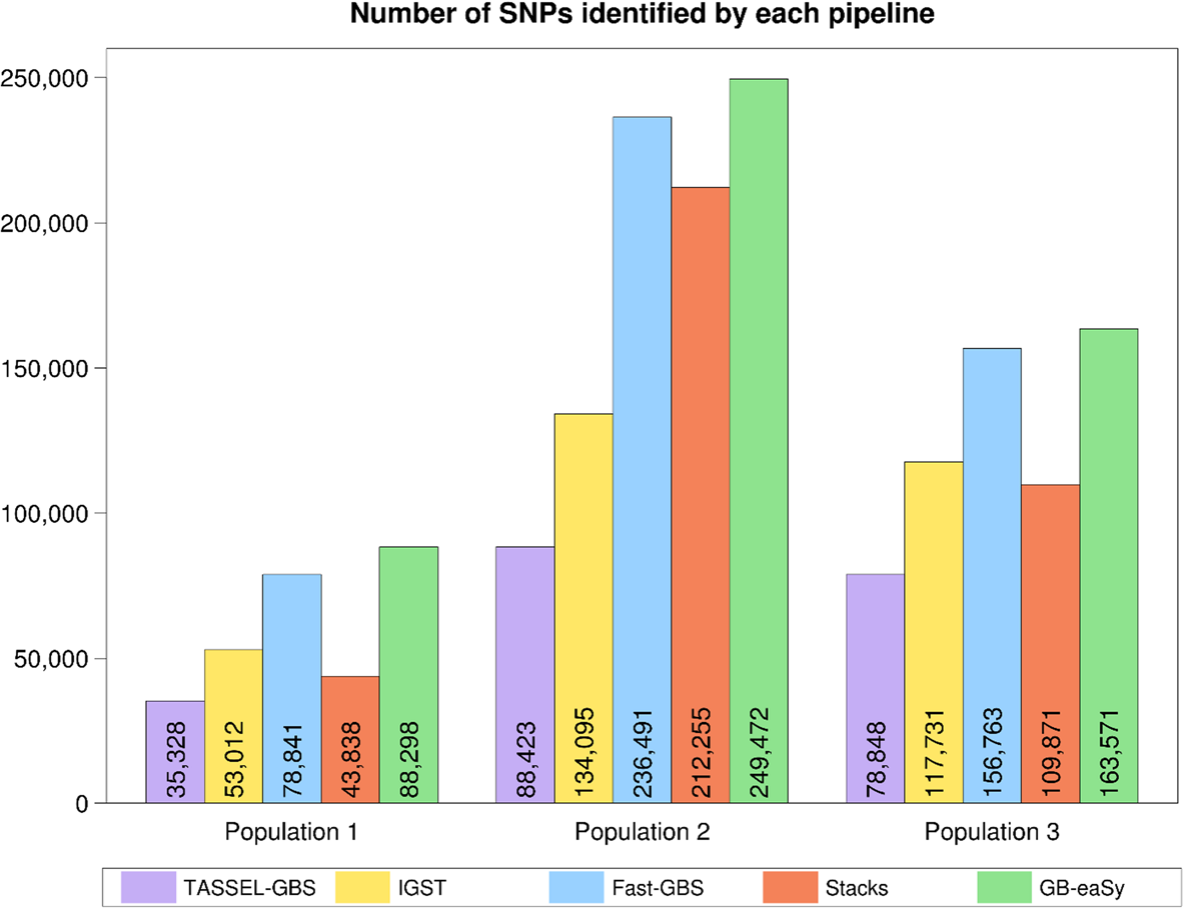
Number of SNPs identified by each pipeline in 3 populations. SNPs with a minimum read depth of 2 reads are shown

**Fig. 2:**
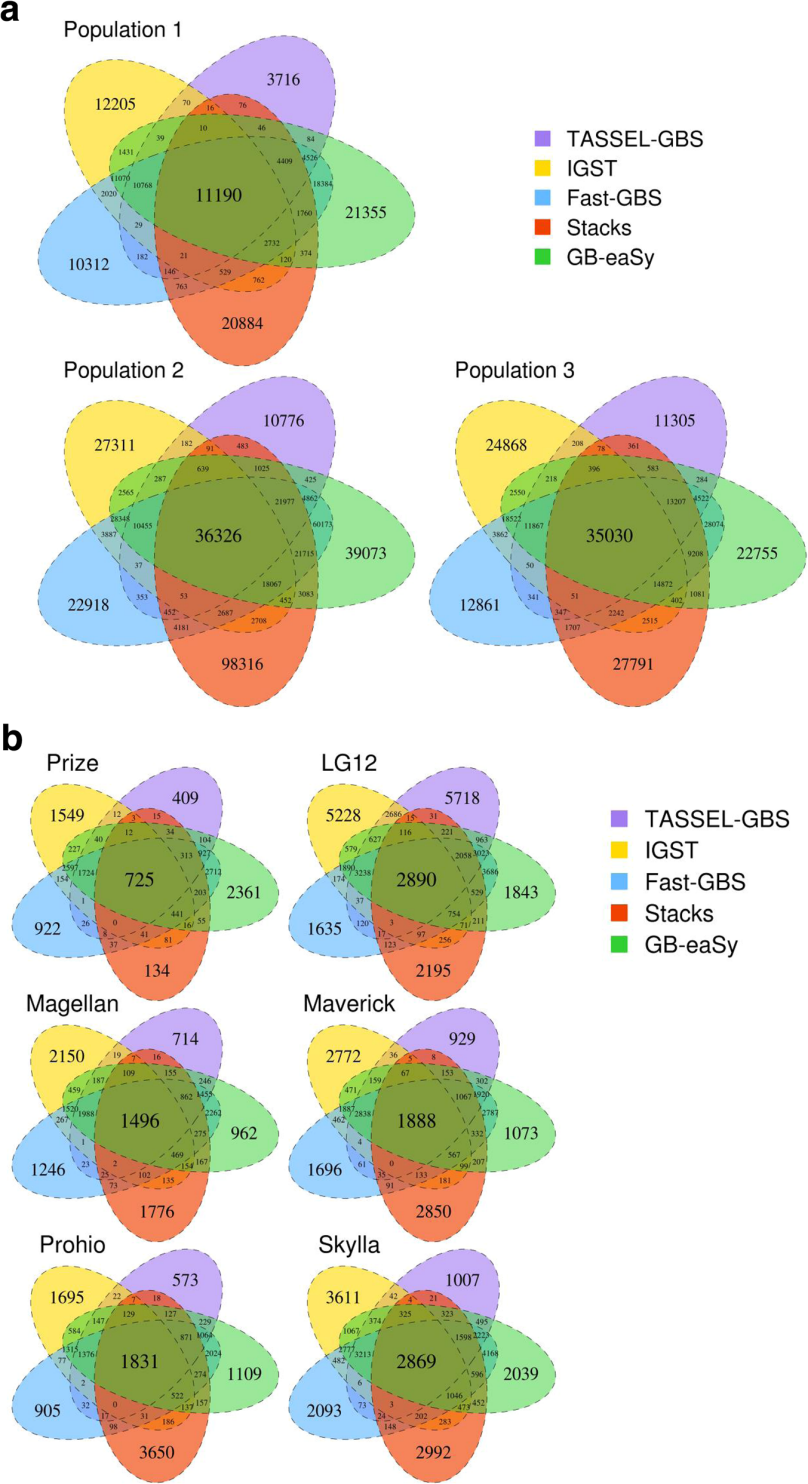
SNP overlap among 5 GBS pipelines. **a** shows overlap for the 3 populations. **b** shows overlap for 6 lines from those populations: Prize is from GBS Population 1, LG12 is from GBS Population 2, and the four remaining lines are from GBS Population 3. SNPs with a minimum read depth of 2 reads are shown. All SNPs were called relative to the Williams 82 reference genome

In terms of SNP yield, the relative ranking of each pipeline remained similar across all three populations: GB-eaSy called the most SNPs, followed in order by Fast-GBS, IGST and Stacks (rank depending on population), and TASSEL-GBS (Fig. 1). In Population 1, the number of SNPs identified ranged from 35,328 (TASSEL-GBS) to 88,298 (GB-eaSy). Population 2 had the greatest number of SNP calls, ranging from 88,423 (TASSEL-GBS) to 249,472 (GB-eaSy); the comparatively large SNP yield of Population 2 likely resulted from the HiSeq4000 outputting 150,000 more reads than the HiSeq2500 used with Populations 1 and 3 (Table 1). In Population 3, the number of SNPs called ranged from 78,848 (TASSEL-GBS) to 163,571 (GB-eaSy). Within each population, a small portion of SNPs was called by all five workflows, with the proportion of convergent SNPs being roughly consistent (Fig. 2a). A similar trend appears in the data for individual soybean lines (Fig. 2b).

**Fig. 3:**
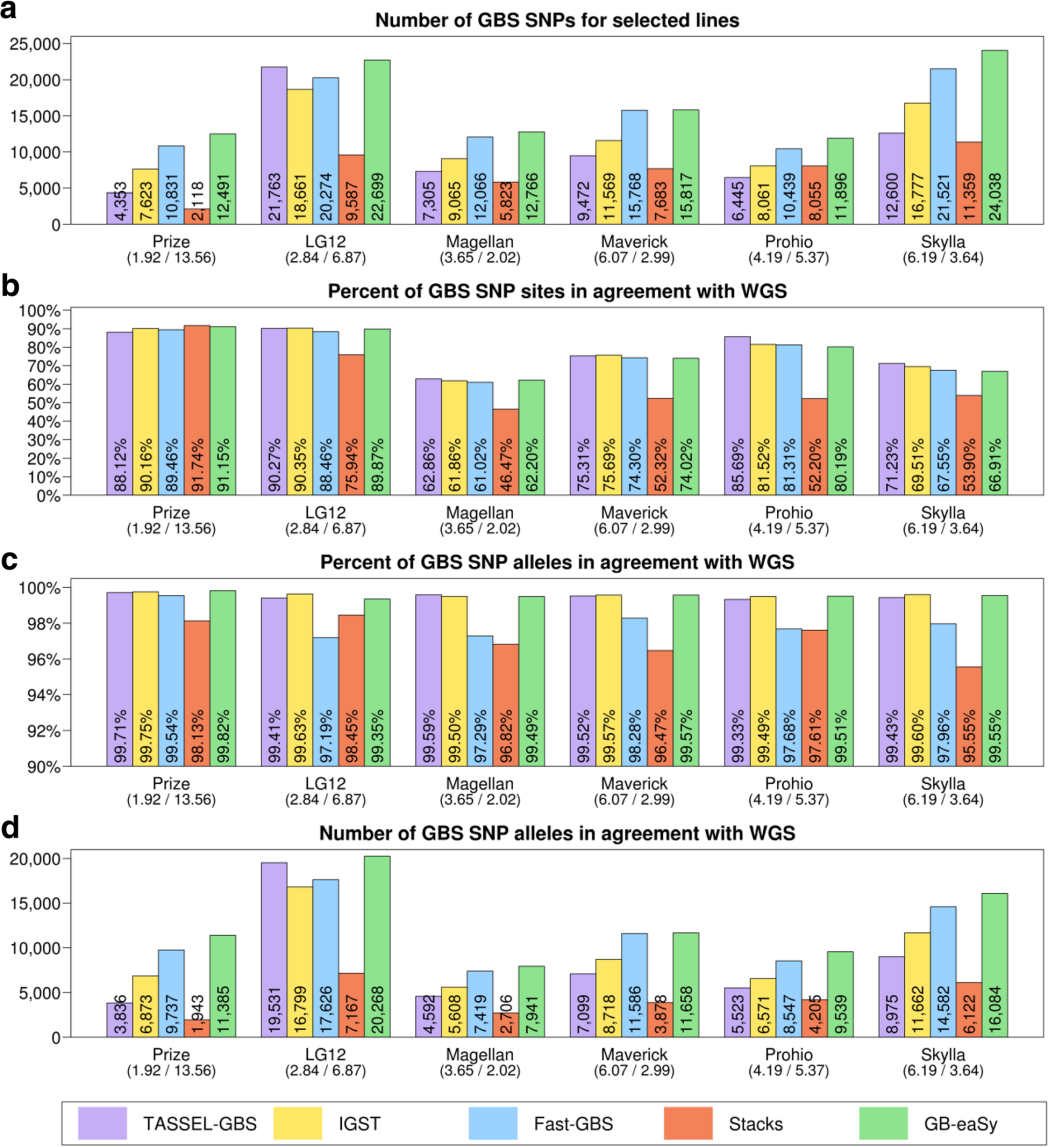
Comparisons between GBS SNPs and WGS SNPs for 6 individual soybean lines. Prize is from GBS Population 1, LG12 is from GBS Population 2, and the four remaining lines are from GBS Population 3. Panel **a** shows the total number of SNPs identified in each line by 5 GBS pipelines. Panel **b** shows the percent of GBS SNP sites from panel A in agreement with WGS for each line. Panels **c** and **d** show the percent and number (respectively) of GBS SNP alleles from panel A in agreement with WGS. SNPs with a minimum read depth of 2 reads are shown. Below each soybean line is shown its average depth of sequenced GBS bases followed by its WGS coverage. All SNPs were called relative to the Williams 82 reference genome

Because the SNP concordance between GBS analysis platforms was unexpectedly low (Fig. 2), whole-genome data of six lines was obtained for comparison of GBS and WGS SNP calls. To avoid biasing these comparisons in favor of a particular GBS platform, GATK HaplotypeCaller (a tool not used by any of the GBS workflows) was used to call SNPs in the WGS datasets. The GBS data for these individual lines follows the population-level pattern of GB-eaSy finding the most GBS SNPs, closely followed by Fast-GBS (Fig. 3a). SNP concordance was calculated as the percentage of GBS SNP sites (e.g. chromosome 1, position 8144) that were also identified by WGS (Fig. 3b). Depending on the line under study, either Stacks, TASSEL-GBS or IGST exhibited the highest SNP concordance with WGS. Across all pipelines, SNP concordance was relatively lower in the lines Magellan, Maverick, Prohio and Skylla due to the low coverage of their WGS data (ranging from 2.02× to 5.37×) and therefore fewer sites sampled (Fig. 3b).

We also assessed the allelic agreement (e.g. chromosome 1, position 8144, nucleotide C) between GBS SNP calls and WGS SNP calls for the set of concordant SNPs identified above (Fig. 3c). In every line examined, GB-eaSy, TASSEL-GBS and IGST all achieved high allelic agreement (above 99%) with WGS, Fast-GBS reached allelic agreement between 97.19% and 99.54%, and Stacks reached allelic agreement between 95.55% and 98.45%. While GB-eaSy, TASSEL-GBS and IGST attained similarly high WGS-agreement rates, GB-eaSy identified the greatest number of SNPs in allelic agreement with WGS in each line (Fig. 3d).

### Missing data

GBS, unlike RAD-seq used for biological diversity analysis, is tuned to identify as many SNPs as possible, with missing data accounted for in later analysis by imputation of haplotypes using reference genome data. However, any GBS data analysis must consider the large proportion of missing/unsampled data, which can often be a limiting factor in downstream applications of the genotype data. The more sensitive a method is to polymorphisms with lower coverage, the more missing data in percentage terms is likely to be observed when comparing samples; therefore, the key parameter is the outright number of SNPs that are present in a sufficient proportion of lines for the analysis to be used. Within the three populations, the average percentage of sampled SNPs not present in any given line was fairly consistent: 83.4% (GB-eaSy) to 89.7% (Stacks) in Population 1, 59.4% (TASSEL-GBS) to 71.5% (GB-eaSy) in Population 2, and 62.4% (TASSEL-GBS) to 69.6% (GB-eaSy) in Population 3 (Table 4). In Population 1, GB-eaSy found the most SNPs present in at least 25% and 50% of sampled lines, while TASSEL-GBS found more SNPs present in at least 75% and 90% of sampled lines (Table 4). In Population 2, Stacks identified the most SNPs present in at least 25% of lines, GB-eaSy identified the most present in at least 50% and 75% of lines, and TASSEL-GBS identified the most SNPs at the 90% level. Finally, in Population 3, Fast-GBS found the greatest number of SNPs present in at least 25% of lines, while GB-eaSy found the greatest number of SNPs at the 50%, 75% and 90% levels. In this case, the variation in performance across the three populations was substantial, but GB-eaSy showed the best or among the best performance for each population. Notably, since each pipeline produces a different subset of valid SNPs (Fig. 2), the optimal strategy for minimizing missing data is likely the combination of multiple approaches.

**Table 4:** Missing data fraction generated by each GBS pipeline

	TASSEL	IGST	Fast-GBS	Stacks	GB-eaSy
Population 1					
Missing data per line	84.5%	85.4%	85.0%	89.7%	83.4%
SNPs in 25% of lines	6812	12,334	18,731	3576	23,633
SNPs in 50% of lines	1237	1714	2984	202	3558
SNPs in 75% of lines	736	112	382	31	407
SNPs in 90% of lines	335	25	75	2	119
Population 2					
Missing data per line	59.4%	70.8%	70.0%	66.1%	71.5%
SNPs in 25% of lines	65,119	68,805	122,801	142,154	120,437
SNPs in 50% of lines	35,107	39,055	76,485	52,991	76,717
SNPs in 75% of lines	2185	1548	4418	372	4880
SNPs in 90% of lines	973	26	219	21	187
Population 3					
Missing data per line	62.4%	69.3%	68.4%	67.2%	69.6%
SNPs in 25% of lines	54,960	65,695	88,904	69,300	88,025
SNPs in 50% of lines	18,859	22,369	32,077	19,756	32,698
SNPs in 75% of lines	6196	7813	12,204	4539	13,005
SNPs in 90% of lines	775	479	934	98	1352

*The average percent of missing data per line is shown, as well as the number of SNPs detected at various proportions within each population*

### Run time and disk space

The pipelines differed widely in their time to completion. TASSEL-GBS (including the initial Cutadapt step) finished most rapidly for each population (Table 5), as expected from its extensive use of tag heuristics to speed alignment. Fast-GBS and GB-eaSy alternately ranked as second and third fastest, depending on the population and the total number of reads. Stacks and IGST used the most wall-clock time per sample, with IGST taking at least three times as long as TASSEL-GBS in every population.

**Table 5:** Wall-clock time to completion for each GBS pipeline (h:mm)

	TASSEL	IGST	Fast-GBS	Stacks	GB-eaSy
Population 1	2:08	12:17	3:20	8:36	5:21
Population 2	4:58	18:46	8:01	16:34	6:51
Population 3	3:38	11:28	4:06	10:15	4:23

The disk space required paralleled the run time in most pipelines (Table 5). For each population, TASSEL-GBS required the least amount of storage. GB-eaSy and Stacks used approximately twice TASSEL-GBS’ disk space requirement. Despite their parameters being set to delete intermediate files where applicable, IGST and Fast-GBS used substantially more disk space than the other methods.

## Discussion

Despite the availability of multiple tools for GBS data processing, a need exists for a GBS pipeline that is easy to install, fits with standard tools, is optimized for high density SNP calling in polyploid crop genomes, and quickly and reliably identifies a large number of accurate SNPs while minimizing its storage footprint. We developed GB-eaSy, a GBS bioinformatics pipeline suitable for both command line novices and experienced bioinformaticians, and aim it primarily at the soybean community, where use of such processing software is increasing. However, GB-eaSy should be applicable to any non-model plant species with a reference genome, particularly to polyploids with repetitive genomes such as soybean. The 1.1-gigabase, recently paleopolyploid soybean genome contains multiple copies of 75% of its genes [18], which presents challenges to accurate processing of genomic data. Therefore, soybean qualifies as a suitable test subject to assess the accuracy of GB-eaSy’s SNP calls. Comparison of GB-eaSy to other GBS data workflows indicated that GB-eaSy rapidly and accurately identified the most SNPs in all three soybean populations examined, without demanding excessive disk space.

### Different SNP calling strategies

A key difference among GBS pipelines that may explain their discrepant results is the software used for variant calling, and its approach to determining the consensus genotype in a group of reads and whether that consensus varies from the reference. Both IGST and GB-eaSy use BCFtools/SAMtools as the variant caller, which relies on a Bayesian strategy to select as the consensus genotype at a given locus the base with the highest Phred score that maximizes the posterior probability [31]. If the consensus genotype at the locus differs from the reference, a SNP is called. Previous work has validated the accuracy of the BWA and SAMtools/BCFtools combination used in IGST and GB-eaSy. For instance, [32] evaluated thirteen variant calling pipelines consisting of combinations of three read aligners (BWA-MEM, Bowtie2, Novoalign) and four variant callers (GATK HaplotypeCaller, SAMtools mpileup, Freebayes, Ion Proton Variant Caller) against a dataset of highly confident “gold standard” human variants published by the 1000 Genomes Project. In that study, the combination of BWA-MEM with SAMtools achieved the greatest accuracy in SNP identification. The two pipelines using these tools in our study (IGST and GB-eaSy) attained the greatest allelic concordance with WGS in the six lines studied.

Each of the other three pipelines investigated here uses a different variant caller. TASSEL-GBS, which calls SNPs using its own binomial likelihood ratio method [16], also agreed well with WGS SNP calls. However, because it found fewer SNPs overall, TASSEL-GBS’ number of validated SNPs was lower than that of GB-eaSy and IGST. Stacks uses a multinomial-based likelihood model for SNP calling, which produced an allelic agreement above 95% but the fewest validated SNPs in each line due in part to its finding fewer SNPs overall. Stacks’ variant caller consults the reference genome only for read placement, not for nucleotide comparisons, as it is optimized for high-coverage analysis of biological diversity RAD sequencing experiments in which reference genomes are often not available [12]. For the low-coverage data typical of plant breeding workflows, it is likely a disadvantage that Stacks does not utilize the Bayesian priors available from high-quality reference genomes. However, for organisms lacking a reference genome, the Stacks approach is likely optimal. Finally, Fast-GBS’ variant caller, Platypus, uses a haplotype-based strategy to identify variants. A previous analysis [33] found that comparison of Fast-GBS SNP calls with WGS data in soybean yielded an accuracy of 98.7%, a result consistent with those presented here. Platypus’ superiority in indel identification but comparatively lower performance in SNP calling has been reported [34], which may explain its slightly lower agreement with WGS compared to the tools used in TASSEL-GBS, IGST and GB-eaSy.

Across all six lines examined, GB-eaSy, TASSEL-GBS and IGST identified SNPs with the greatest accuracy (over 99%), based on comparison to WGS SNPs called by GATK HaplotypeCaller (Fig. 3). The accuracy of Fast-GBS and Stacks was lower but still reasonably high (never below 97%). This high accuracy among all five workflows, coupled with the low SNP convergence between them, indicates that they arrived at largely complementary sets of valid SNP calls (Fig. 2b and Fig. 3). For instance, GB-eaSy, TASSEL-GBS and IGST converged on just 2501 (12.85%) of their total 19,465 unique SNPs found in Prize (Fig. 2b). Similarly, these three pipelines converged on just 6781 (17.02%) of their 39,853 unique SNPs found in Skylla (Fig. 2b). These results echo a previous report on barley GBS data in which approximately half of SNPs called by TASSEL-GBS and BCFtools/SAMtools were unique to each pipeline [35].

### Storage, run time and ease of use

TASSEL-GBS, the workflow with the smallest storage requirements, used approximately half of the hard disk space required by Stacks and GB-eaSy. While it used the least disk space, TASSEL-GBS identified the fewest SNPs. Both IGST and Fast-GBS found more SNPs than TASSEL-GBS but required the largest amount of disk space due to their generation of many uncompressed intermediate files, even with parameters set to delete intermediate files where applicable. This characteristic could hinder their adoption by users with limited computer storage capacity. Across pipelines, these patterns also emerged in run time differences, which may be determined to a large extent by read-write rather than CPU operations. IGST and Stacks required considerably more time to run than TASSEL-GBS, Fast-GBS and GB-eaSy. For instance, IGST needed over 18 h to process data from Population 2, while TASSEL-GBS finished in less than 5 h. Long completion times limit the throughput of data processing, making the slower pipelines less suitable for time-sensitive projects. GB-eaSy’s run times were intermediate, ranking ahead of IGST, Stacks and occasionally Fast-GBS but behind TASSEL-GBS.

Given the complexities of GBS analysis, a critical element of any bioinformatics pipeline is ease of use. The five analysis platforms in this study rely on two command input strategies. In TASSEL-GBS and Stacks, the user inputs individual commands that each run a different step of the pipeline. In contrast, IGST, Fast-GBS and GB-eaSy automate this process by requiring just one command from the user to execute all steps; however, IGST and Fast-GBS also depend on adherence to a rigid convention for file naming and directory structure to ensure successful completion. GB-eaSy does not require the user to follow strict instructions for setting up directory structure or naming files. Instead, it uses a parameters file to customize the analysis for each project based on user input.

Another consideration in ease of use is the ability of a method to carry out all the steps necessary to produce accurate SNP calls. For our data, TASSEL-GBS and Fast-GBS required extra steps not built into their pipelines to improve the accuracy of their SNP calls. Fast-GBS initially appeared to identify significantly fewer SNPs than the other methods and showed lower agreement with WGS. However, after decomposition of compound SNPs into allelic primitives using the VariantsToAllelicPrimitives script, the apparent performance of Fast-GBS improved considerably; these optimized results were used in the comparison. Prior to running TASSEL, we removed adapter sequence from the reads using Cutadapt, adding an additional step to the workflow, after finding that adapter contamination significantly impaired the accuracy of TASSEL-GBS SNP calls. Again, the optimized results after the trimming step were used in the comparison. In GB-eaSy, these additional steps either are not required or are built into the pipeline itself.

## Conclusions

Here we introduce the GB-eaSy pipeline and compared its performance to four other GBS workflows and to whole-genome sequencing on low-coverage data from soybean. Differences were apparent between the performance of these methods depending on the aims of the developers. TASSEL-GBS was designed for plant breeding applications and to run on individual PCs, and is thus optimized for maximum computational efficiency. The compromises inherent in the tag strategy limit the number of SNPs that TASSEL-GBS can identify using datasets such as those utilized here. Stacks is a method developed primarily for high-depth RAD sequencing on organisms without reference genomes. It is likely to be an excellent choice for breeders in orphan crops, as well as for biological diversity applications, but the reference-genome independence of the variant calling algorithm and the low-coverage data used here render the current version less accurate than methods incorporating reference sequences for low-depth GBS in soybean. Fast-GBS and IGST are, like GB-easy, methods designed for plant breeding applications on complex crops with high-quality reference genomes. The overall performance of these methods in terms of SNP number and accuracy is similar. GB-easy has an advantage over the other methods in terms of resources needed (particularly disk space), ease of implementation, and number of accurate SNPs identified. Although our results demonstrate relatively low SNP concordance between GBS pipelines, comparison of each GBS pipeline to WGS data indicates that the SNP calls from each are highly accurate, particularly those generated by GB-eaSy, TASSEL-GBS and IGST. These findings suggest that a comprehensive approach integrating the results from multiple GBS analysis methods may be the optimal strategy to obtain the largest, most highly accurate SNP yield possible from low-coverage polyploid sequence data.

## Abbreviations

GBS: Genotyping-by-sequencing
Indel: Insertion-deletion
RAD: Restriction site associated DNA
SNP: Single-nucleotide polymorphism
WGS: Whole-genome sequencing
